# A Multidimensional Comparison of Psychosocial Adjustment, Functional Hearing, and Device Benefit in Adult Hearing Device Users

**DOI:** 10.3390/healthcare14131980

**Published:** 2026-07-03

**Authors:** Bamini Gopinath, Alicia Zou, Jessica Turner, George Burlutsky, Diana Tang

**Affiliations:** Macquarie University Hearing Research Centre, School of Health Sciences and Nursing, Macquarie University, Sydney, NSW 2109, Australia; alicia.zou@mq.edu.au (A.Z.); jessica.turner@mq.edu.au (J.T.); george.burlutsky@mq.edu.au (G.B.); d.tang@mq.edu.au (D.T.)

**Keywords:** hearing loss, hearing aid, cochlear implant, adults, outcome evaluation

## Abstract

**Background**: While previous research has examined individual aspects of hearing loss, such as perceived hearing handicap, functional hearing ability, or hearing device outcomes, these domains are rarely evaluated together within the same cohort. We aimed to address this gap by providing a multidimensional assessment of hearing-related outcomes and comparing measures of perceived hearing handicap, psychosocial adjustment to hearing loss, functional hearing ability, and device-related benefit between hearing aid, cochlear implant, and bimodal device users. **Methods**: Cross-sectional data were collected from 749 adults aged ≥40 years using hearing aids (n = 467), cochlear implants (n = 150), or bimodal devices (n = 132), during 2022–2024. Measures of hearing handicap (Hearing Handicap Inventory for the Elderly Screening, HHIE-S), psychosocial responses to hearing loss (Attitudes towards Loss of Hearing Questionnaire, ALHQ), real-world hearing ability (Speech, Spatial and Qualities of Hearing Scale, SSQ), and outcomes of hearing device use (International Outcome Inventory for Hearing Devices, IOI-HD) were collected. **Results**: After multivariate adjustment, hearing aid users versus bimodal and cochlear implant users had significantly higher ALHQ scores in the domain of negative associations (suggestive of negative attitudes or stigma attached to hearing aid use): 2.22 (0.05) versus 1.92 (0.09) (*p* = 0.0067) and 1.90 (0.09) (*p* = 0.0145), respectively. Hearing aid users had significantly higher SSQ spatial scores (better sound localization and spatial perception) compared to bimodal device users (5.13 versus 4.45, *p* = 0.0254). Total IOI-HD scores were significantly lower in hearing aid users (reflective of irregular, inconsistent daily usage of the device) compared to cochlear implant users (*p* < 0.0001) and bimodal device users (*p* = 0.0007). HHIE-S scores were not significantly different between device user groups after multivariate adjustment. **Conclusions**: This multidimensional approach provides new insight into the heterogeneity of hearing experiences and may help inform more person-centered models of hearing care.

## 1. Introduction

Globally, 1.5 billion people have hearing loss, and this prevalence is expected to increase due to aging populations [1]. Without treatment, hearing loss can negatively impact communication, which results in further consequences, including poorer psychosocial health outcomes [2,3,4] increased dementia risk [5] and mortality [6]. Hearing devices, including hearing aids and/or cochlear implants, are often recommended for people with hearing loss who meet certain criteria. For hearing aids, which serve to amplify sound, candidacy is objectively determined by a hearing assessment and may be complemented by a needs assessment to understand the individual context. For cochlear implants, which use neural activity to make sounds audible and speech understandable, the 60/60 guidelines suggest that cochlear implant candidacy evaluation is recommended if an individual presents with a pure-tone average of ≥60 dB HL in the better ear and an unaided monosyllabic word score of ≤60% correct in the better ear [7].

While users of these hearing devices typically have different levels of hearing severity—hearing aid users often have mild-to-moderate hearing loss, and cochlear implant users tend to have severe or profound hearing loss—the desired outcomes or benefits from their hearing intervention are more aligned. Recently, umbrella reviews were conducted to evaluate the benefits of both hearing devices [8,9]. A number of potential benefits were associated with hearing aid use—speech perception, communication function, hearing handicap, and self-assessed hearing aid benefit—but high-quality evidence derived from robust study designs was lacking [9]. While there was more evidence for cochlear implants and a general acknowledgment that they appear beneficial, there was a similar need for more high-quality evidence to thoroughly understand the outcomes in adults.

In children, the Longitudinal Outcomes of Children with Hearing Impairment (LOCHI) study is a population-based, prospective study that has been providing meaningful insights to evaluate the effectiveness and cost-effectiveness of early intervention in a population of children in Australia [10]. It comprehensively captures prospective data on a range of predictors and outcomes associated with hearing aid and/or cochlear implant usage over multiple time points, with ongoing follow-up. A LOCHI-equivalent in adults is needed to understand the effectiveness of early intervention in a population where hearing device uptake is underwhelming; 11% of adults with hearing loss own and use a hearing aid [11], and less than 10% of adults who could benefit from a cochlear implant have acquired one [12].

The Hearing Impairment in Adults: a Longitudinal Outcomes Study (HALOS) [13] seeks to address the gaps in knowledge about the impact and effectiveness of hearing aids and cochlear implants in adults. As the first wave of follow-up is currently underway, this paper aims to present cross-sectional data on hearing-related outcomes in this cohort of adults aged 40+ years, including: (a) Self-perceived hearing handicap (Hearing Handicap Inventory for the Elderly Screening, HHIE-S); (b) Psychosocial adjustment to hearing loss (Attitudes towards Loss of Hearing Questionnaire, ALHQ); (c) Self-reported hearing ability (Speech, Spatial and Qualities of Hearing Scale, SSQ); and (d) Benefits of hearing devices (International Outcome Inventory for Hearing Devices, IOI-HD). By examining these complementary domains together, this study offers new insights into the heterogeneity of lived hearing experiences and the interplay between functional hearing, psychological adaptation, and perceived benefits of hearing devices in adults.

## 2. Materials and Methods

### 2.1. Recruitment of Participants

This study was approved by the Macquarie University Human Research Ethics Committee (ID: 11262) and Southern Adelaide Local Health Network (ID: LNR/22/SAC/88). All methods were carried out in accordance with relevant guidelines and regulations. Participants were recruited as part of the HALOS project, which collects cross-sectional and longitudinal survey data on a range of health and social outcomes in adults aged 40 years and older who use a hearing aid and/or cochlear implant [13]. Recruitment flyers were disseminated widely through various hearing service providers, consumer support groups, and existing University platforms, including volunteer databases, websites, and social media.

### 2.2. Data Collection

Participants self-completed an online survey via Research Electronic Data Capture (REDCap) version 17.x or a paper-based survey via post or email within a two-week period. Participants took 60–75 min on average to complete the survey. Written informed consent was obtained from all participants prior to survey commencement.

### 2.3. Study Factors

Participants were categorized based on self-reported hearing device usage, including hearing aid users, cochlear implant users, and bimodal users. Data on age, sex, and duration of device use (number of years) were collected and adjusted for in the current analyses. For pure-tone audiometry, the most recent audiometric data were obtained for participants through their authorized consent to release this information from their hearing service provider. This occurred via the study team providing participating hearing service providers with an aggregated list of participant names, which were sourced from their clinic(s). The collected data included audiometric thresholds for air- and bone-conduction stimuli in both ears for octave frequencies at 0.25 to 8.0 kHz and/or four-frequency average hearing loss (4FAHL) data, measured at 0.5, 1, 2, and 4 kHz. This helped to determine the severity of hearing loss in participants.

Health-related quality of life was assessed using the Health Utilities Index Mark 3 (HUI-3), a preference-based instrument that generates utility scores from multiple health domains, including hearing, vision, and mobility [14]. These variables were considered as potential covariates in the statistical analyses.

### 2.4. Outcome Measures

Subjective hearing loss or perceived hearing difficulty was measured using the 10-item HHIE-S. The HHIE-S has been validated for screening hearing impairment in older adults and was originally developed to assess perceived functional limitations associated with hearing impairment [15,16]. HHIE-S consists of five social or situational items and five emotional response items with a scoring of 4 points for “yes”, 2 points for “sometimes”, and 0 points for “no” responses. A total HHIE-S score of ≥8 indicates the presence of a hearing handicap, whereby a score of 10 to 24 is indicative of mild to moderate handicap, and >26 is indicative of severe handicap [16].

The 12-item Speech, Spatial, and Qualities Hearing Scale (SSQ12) [17] was used to evaluate hearing disability across nine subscales—speech in noise, speech in speech, multiple speech streams, localization, distance and movement, segregation, identification of sound, quality and naturalness, and listening effort. Items mentioning ‘hearing aid/s’ have been replaced with ‘hearing device(s)’ to include cochlear implant users. Response options for each question were presented on an 11-point Likert scale ranging from 0 to 10, with higher scores indicating greater hearing ability. The International Outcome Inventory for Hearing Aids (IOI-HA) [18] is a seven-item instrument used to assess the effectiveness of hearing aids. Each item reflects a particular domain, which includes daily use, benefit, residual activity limitations, satisfaction, residual participation restrictions, impact on others, and quality of life. The IOI-HA has been modified to include cochlear implant users by replacing the use of “hearing aid(s)” with “hearing device(s)”. This instrument will be referred to as the International Outcome Inventory for Hearing Devices (IOI-HD). As with the IOI-HA, the IOI-HD includes a 5-point Likert scale for each item.

The Attitudes towards Loss of Hearing Questionnaire v2.1 (ALHQ v2.1) [19] is an improved version of Saunders and Cienkowski’s original ALHQ. As with the IOI-HA, the ALHQ v2.1 was modified to include cochlear implant users by replacing the use of “hearing aid(s)” with “hearing device(s)”. This 22-item questionnaire evaluates attitudes towards hearing loss and hearing aids across five domains: denial of hearing loss, negative associations, negative coping strategies, manual dexterity and vision, and hearing-related esteem. Denial of hearing loss includes two items (Question 1 and 2) that are reverse-scored. For ease of interpretation, a high score on each scale is associated with a “negative” attitude; that is, denial rather than acceptance of hearing loss, negative associations with hearing devices, poor coping strategies, poor manual dexterity and/or visual acuity, and low hearing-related esteem. Thus, low ALHQ scores are preferable to high ALHQ scores in terms of better outcomes.

### 2.5. Data Analysis

There were three groups (hearing aids, bimodal devices, and cochlear implants) in our study. General Linear Models (proc GLM, SAS 9.4) were used to provide analysis of variance (ANOVA) and analysis of covariance (ANCOVA) for our study. To determine whether there was a significant difference between the means of these 3 groups, we used ANOVA for models without covariates (Table 1). This was done using the F-test. The same approach was used for ANCOVA.

ANCOVA was used to examine associations between hearing device type (hearing aids, cochlear implants, bimodal devices) and the primary hearing-related outcomes—HHIE-S, SSQ, ALHQ, and IOI-HD scores. Covariates included age, sex, number of years of device use, severity of hearing loss (4FAHL), and total HUI-3 scores. Least squares means were calculated to compare adjusted group means, and post hoc pairwise comparisons were conducted with *p*-values presented. Results are presented as adjusted group means (standard error). Effect sizes across the three hearing device groups were estimated using Eta-Squared (ANOVA) and Partial Eta-Squared (ANCOVA).

A Bonferroni correction was applied to the ANCOVA results to account for multiple testing (Table 1). To control the family-wise error rate due to multiple comparisons, Bonferroni-adjusted *p*-values were used for all pairwise comparisons among groups. The analyses were performed using the Bonferroni option in SAS PROC GLM, which adjusts *p*-values for multiple pairwise differences following the ANCOVA. Statistical significance was set at *p* < 0.05. All analyses were performed using SAS Version 9.

## 3. Results

A total of 749 participants with complete data were included in the analyses: 467 hearing aid users, 132 bimodal device users, and 150 cochlear implant users. Within the hearing aid group, there were 46 unilateral and 421 bilateral device users. In the cochlear implant group, there were 64 unilateral and 86 bilateral device users. Table 1 presents demographic, hearing, and psychosocial characteristics of the sample stratified by device group. Significant differences in age, ALHQ (psychosocial adjustment to hearing loss), SSQ (self-reported hearing ability), and IOI-HD (device-related benefit) scores were observed across the three device-user groups. A higher proportion of cochlear implant and bimodal device users had moderate-to-severe hearing loss (^3^40 dB HL) than hearing aid users (Table 1). Additionally, the majority of cochlear implant and bimodal device users were using their devices for 10+ years, compared with hearing aid users (Table 1).

Table 2 shows that for the HHIE-S, after adjusting for age and sex, hearing aid users compared to bimodal users had significantly lower total [21.19 (0.45) versus 23.37 (0.83), *p* = 0.0223]; and social sub-scale scores [12.99 (0.23) versus 14.36 (0.43), *p* = 0.0055]. However, after multivariate adjustment, significant differences in HHIE-S scores did not persist. All three device users had total HHIE-S scores > 20, indicating moderate-to-severe self-perceived hearing handicap, which indicates that they are still experiencing substantial social and emotional consequences due to hearing difficulties.

Total ALHQ scores were significantly higher in hearing aid users (2.43 (0.02)) compared to bimodal device users (2.30 (0.03); *p* = 0.0038) after multivariate adjustment (Table 3). Similarly, hearing aid users versus bimodal and cochlear implant users had significantly higher ALHQ scores in the domain of negative associations (suggestive of negative attitudes or stigma attached to hearing aid use), after adjusting for all potential confounders: 2.22 (0.05) versus 1.92 (0.09) (*p* = 0.0067) and 1.90 (0.09) (*p* = 0.0145), respectively. After multivariate adjustment, hearing aid users also had higher scores compared to bimodal device users in the ALHQ domain of hearing-related esteem (indicating more negative self-esteem linked to hearing loss): 3.15 (0.05) versus 2.84 (0.10) (*p* = 0.0199; Table 3).

After multivariate adjustment, hearing aid users compared with bimodal device users had significantly higher SSQ spatial scores (Table 4). This indicates that hearing aid users had better sound localization and spatial perception compared to bimodal users [5.13 (0.12) versus 4.45 (0.23), *p* = 0.0254]. Furthermore, all three device user groups had total SSQ scores below 8, suggesting that these individuals are likely to be experiencing substantial difficulties in their daily listening situations.

Total IOI-HD scores were significantly lower in hearing aid users (reflective of irregular, inconsistent daily usage of the device) compared to cochlear implant users [27.31 (0.22) versus 29.35 (0.40), *p* < 0.0001] and bimodal device users [27.31 (0.22) versus 29.03 (0.41), *p* = 0.0007] (Table 5). Similarly, after multivariate adjustment, hearing aid users had significantly lower IOI-HD Factor 1 scores (covers hours of use, benefit, satisfaction, and quality of life—better outcome) compared to cochlear implant users [16.34 (0.15) versus 18.00 (0.27), *p* < 0.0001] and bimodal device users [16.34 (0.15) versus 17.65 (0.27), *p* < 0.0001].

## 4. Discussion

This study is novel in that it provides a multidimensional assessment of hearing-related outcomes in adults aged 40+ years by determining measures of perceived hearing handicap, psychosocial adjustment to hearing loss, functional hearing ability, and device-related benefit within the same cohort of device users. Hearing aid users, compared with both bimodal and cochlear implant users, had significantly worse outcomes related to ALHQ (psychosocial adjustment to hearing loss) and IOI-HD (device usage benefits) measures. Conversely, hearing aid users, versus bimodal device users, had better outcomes related to SSQ measures (functional hearing ability). After accounting for all potential confounders, no significant differences in self-perceived hearing handicap (HHIE-S scores) were observed between the device user groups.

In our study, HHIE-S scores were not significantly different between the three hearing device user groups once confounders were considered, indicating similar levels of self-perceived hearing handicap. However, it is important to note that hearing aid, cochlear implant, and bimodal device users had mean total HHIE-S scores > 20, indicating moderate-to-severe self-perceived hearing handicap; hence, they are likely to still be experiencing substantial social and emotional consequences due to hearing difficulties. This contrasts with findings from a US study of 115 hearing aid and cochlear implant users, which found mean HHIE-S scores reduced significantly to <20 (mild hearing handicap), five years after device provision [20]. Most hearing device users in HALOS had been using their device for more than five years, and while device usage may be longer in these participants, it does not necessarily equate to optimal support over those years. That is, people can develop new barriers (e.g., cognitive load changes, social role changes, device dissatisfaction, and reduced use in noise) that keep HHIE-S scores elevated. Further, there may be service delivery differences between the Australian HALOS and the US study, such as ongoing aural rehabilitation, follow-up, and counseling intensity, access to accessories/upgrades, that could account for differences in HHIE-S scores between the cohorts. Ultimately, our findings of greater substantial residual participation restriction and psychosocial burden in long-term hearing device users highlight the need for ongoing, multidisciplinary post-fitting/implant rehabilitation and systematic review pathways, rather than reliance on device provision alone [21].

There is a dearth of studies that have compared ALHQ scores between hearing device users. Hence, HALOS contributes to the evidence base by showing that hearing aid users reported a consistently more negative psychosocial profile around hearing loss and device use than cochlear implant and bimodal device users. The pattern is especially clear in the ALHQ domain of negative associations, as hearing aid users appeared to endorse more stigma-related or negative beliefs about hearing devices than both comparison groups. This implies that for many hearing aid users, the device may serve as a more salient symbol of disability or aging, shaping day-to-day self-perceptions and social comfort, even when functional outcomes may be acceptable. Moreover, the elevated hearing-related esteem score in hearing aid versus bimodal device users reinforces that this may not only be about external stigma, but also internalized impact; that is, potentially more self-doubt, reduced confidence, and/or diminished identity/esteem linked to hearing loss [22]. However, this was not directly measured or assessed in this study and would require further confirmation by other studies.

Observed differences in ALHQ scores between the device user groups, therefore, likely reflect differences in identity, care pathways, and expectation management rather than device effects alone. For instance, cochlear implant users typically undergo more intensive counseling and structured rehabilitation, which may facilitate acceptance and more realistic expectations, supporting better psychosocial adjustment [21]. Consistent with response shift theory, this process may recalibrate internal standards and promote more favorable self-appraisal despite ongoing impairment in cochlear implant users [23]. By comparison, hearing aid users may experience greater expectation–outcome mismatch, particularly in challenging listening environments, leading to frustration, reduced self-efficacy, and reinforcement of negative beliefs about device benefit [24].

Hearing aid users demonstrated significantly better spatial hearing than bimodal device users (i.e., higher adjusted mean SSQ spatial scores. Despite these relative advantages in hearing aid users, total SSQ scores below 8 across all groups indicate persistent, clinically meaningful listening difficulties in everyday environments, particularly in complex or noisy settings [25]. This aligns with evidence that both hearing aids and cochlear implants, while improving audibility, do not fully restore normal auditory function, especially for higher-order tasks such as spatial hearing and speech understanding in noise [26,27]. Together, these findings highlight that while hearing aids may better preserve spatial cues, substantial unmet needs in real-world listening remain across all device users.

Hearing aid users demonstrated significantly lower total IOI-HD and Factor 1 scores than both cochlear implant and bimodal users, indicating less consistent use and lower perceived benefit and satisfaction. This is consistent with knowledge that hearing aid users frequently experience more variable benefit, particularly in complex listening environments, which may attenuate satisfaction and reduce consistent usage despite measurable improvements [28]. In contrast, cochlear implantation is typically undertaken in individuals with more severe hearing loss for whom the device provides a substantial and often transformative gain in auditory function, thereby strengthening perceived benefit and reinforcing sustained use [8].

In interpreting these results, it is important to consider effect sizes in addition to statistical significance. In a cohort of this size, small between-group differences can yield statistically significant *p*-values; however, the magnitude of observed effects indicates that several differences were relatively modest. Across outcomes, the eta-squared/partial eta-squared estimates were generally in the small-to-moderate range, indicating that the hearing device group accounted for only a limited proportion of variance in psychosocial adjustment, functional hearing, and device-related benefit. Therefore, while the direction of findings is clinically informative, particularly the more negative psychosocial profile and lower perceived benefit/consistency of use among hearing aid users relative to cochlear implant and/or bimodal users, these differences should not be interpreted as determinative at the individual patient level. Rather, they highlight group-level patterns that may help clinicians identify domains (e.g., attitudes and expectations) where additional support is needed, while recognizing substantial overlap in experiences and outcomes across device groups.

Clinically, findings from this study support a growing evidence base that assuming device provision alone is insufficient for long-term hearing care. For instance, a recent study that pooled analysis of 61,089 adults with hearing loss from 33 countries found that hearing aid provision alone is insufficient and that quality hearing rehabilitation may be the key exposure for dementia prevention in adults with hearing loss [29,30]. In the present study, hearing aid users reported poorer psychosocial adjustment (higher ALHQ) and lower perceived device benefit/consistency of use (lower IOI-HD), despite relatively better spatial hearing (higher SSQ scores). This pattern, from our research and others, suggests a need for more proactive, structured, and holistic hearing care to support sustained daily use and real-world benefit. Further, alternate strategies such as mobile auditory training have been shown to improve word and speech perception performance, adherence, and compliance with hearing aids [31]. For cochlear implant recipients, comparatively better psychosocial and device-benefit outcomes align with maintaining the elements typically embedded in implant pathways, e.g., structured rehabilitation within longer-term review protocols to preserve engagement and address evolving listening goals and challenges over time [21,32]. Therefore, these are areas of focus for future longitudinal cohort studies or randomized controlled studies.

There are several key study strengths, including a large sample of hearing device users recruited from across the country and the availability of robust data on a range of hearing, health, and social outcomes that could be accounted for in the final multivariate analysis. We also used validated instruments to assess key hearing-related outcome measures. Nevertheless, this study is not without limitations. First, this is a cross-sectional study design, and this precludes causal inference. Namely, differences in SSQ, ALHQ, and IOI-HD scores observed between device groups should be interpreted as associations only, not as evidence that specific device use caused those outcomes. The design also cannot capture adaptation processes (for example, whether people psychologically adjust over time after commencing or switching devices), so group differences may reflect pre-existing characteristics, timing since uptake, or self-selection. Consequently, longitudinal data are needed to evaluate the direction of observed relationships and whether device-related differences in key outcomes are sustained over time. Additionally, although we adjusted for a range of measured covariates, residual confounding remains a key limitation given the marked age imbalance between groups, with the hearing aid users substantially older than the cochlear implant and bimodal cohorts. Statistical adjustment may not fully account for age-related differences that are difficult to capture with available measures, including heterogeneity in cognitive reserve, comorbidity burden, and social participation, all of which can shape patient-reported psychosocial outcomes independently of device type. Residual confounding may also arise from factors that were not measured or measured imprecisely, such as duration and severity of hearing deprivation prior to intervention, and rehabilitation intensity. Similarly, socioeconomic factors (for example, health literacy, access to services, and support networks) may differ systematically across device groups and influence expectations, coping strategies, and reported benefits. Consequently, some observed between-group differences in patient-reported outcomes may reflect underlying differences in participant characteristics rather than true device-related effects, underscoring the need for more granular measurement and longitudinal designs to better isolate device-specific associations. Finally, selection bias is possible because participants were recruited via hearing service providers and through voluntary responses to study advertisements (e.g., flyers distributed through service providers). This recruitment approach may preferentially capture individuals who are more motivated or engaged with their hearing care, and who may have different experiences than less-engaged users or those with poorer access to services. As a result, the observed differences in outcomes may not be fully representative of the broader population of hearing device users, potentially limiting generalizability.

## 5. Conclusions

In conclusion, in this large cohort of adults aged 40 years and older who use hearing aids, cochlear implants, or bimodal devices, a multidimensional assessment demonstrated meaningful differences across psychosocial adjustment, functional hearing, and perceived device benefit across the three different device-user groups. Collectively, these findings highlight the heterogeneity of lived hearing experience and reinforce that optimizing outcomes likely requires person-centered, ongoing rehabilitation and psychosocial support alongside device provision, particularly for hearing aid users for whom attitudes and self-perceptions may be key barriers to satisfaction and sustained benefit.

## Figures and Tables

**Table 1:** Characteristics of study participants (n = 749).

Characteristics	Hearing Aid	Cochlear Implant	Bimodal	*p* Value
	(n = 467)	(n = 150)	(n = 132)	
Age (years), Mean (SD)	72.1 (10.0)	63.3 (12.3)	67.2 (11.9)	<0.0001
Male, n (%)	185 (39.6%)	65 (43.3%)	62 (47.0%)	0.2854
HUI-3, Mean (SD)	0.57 (0.24)	0.64 (0.21)	0.58 (0.23)	0.1080
HHIE-S Total Score	21.1 (9.69)	21.1 (9.4)	23.5 (8.8)	0.3480
ALHQ Denial Score	2.09 (0.66)	2.07 (0.64)	1.79 (0.53)	<0.0001
ALHQ Negative Association Score	2.20 (1.00)	1.83 (0.87)	1.87 (0.86)	<0.0001
ALHQ Negative Coping Score	2.91 (0.74)	2.92 (0.76)	3.04 (0.69)	1.0000
ALHQ Dexterity and Vision Score	2.05 (0.99)	1.63 (0.87)	1.69 (0.90)	<0.0001
ALHQ Esteem Score	3.20 (1.09)	3.04 (1.06)	2.72 (1.06)	<0.0001
ALHQ Total Score	2.46 (0.38)	2.30 (0.35)	2.28 (0.35)	<0.0001
SSQ Total Score	4.68 (1.94)	4.31 (2.10)	3.86 (1.84)	<0.0001
IOI-HD Total Score	27.2 (4.8)	30.0 (3.8)	29.3 (4.1)	<0.0001
Hearing device use (years)				
<5	191 (41.0%)	16 (10.8%)	13 (9.8%)	<0.0001
5 ≤ x <10	99 (21.2%)	17 (11.5%)	12 (9.1%)	
≥10	176 (37.8%)	115 (77.7%)	107 (81.1%)	
Hearing loss severity				
≥40 dB HL	274 (67.8%)	112 (82.4%)	116 (95.1%)	<0.0001

HUI-3, Health Utilities Index Mark 3; HHIE-S, Hearing Handicap Inventory for the Elderly Screening; ALHQ, Attitudes towards Loss of Hearing Questionnaire; SSQ, Speech, Spatial and Qualities of Hearing Scale; IOI-HD, International Outcome Inventory for Hearing Devices.

**Table 2:** Adjusted comparison of mean Hearing Handicap Inventory for the Elderly Screening (HHIE-S) scores among hearing device users.

	Device User Groups			
Adjusted Mean Scores (Standard Error)	Hearing Aid	Cochlear Implant	Bimodal	Effect Size (Confidence Level)
**Total Score**				
Model 1	21.19 (0.45)	20.72 (0.80)	23.37 (0.83)	0.010 (0.000, 0.027)
*p*-value ^a^		N.S.	0.0669	
Model 2	21.73 (0.44)	20.77 (0.81)	22.14 (0.83)	0.017 (0.002, 0.039)
*p*-value ^a^		N.S.	N.S.	
**Emotional Score**				
Model 1	8.21 (0.25)	7.59 (0.44)	9.04 (0.46)	0.007 (0.000, 0.022)
*p*-value ^a^		N.S.	N.S.	
Model 2	8.41 (0.25)	7.87 (0.46)	8.65 (0.47)	0.012 (0.000, 0.032)
*p*-value ^a^		N.S.	N.S.	
**Social Score**				
Model 1	12.99 (0.23)	13.14 (0.41)	14.36 (0.43)	0.011 (0.000, 0.029)
*p*-value ^a^		N.S.	0.0165	
Model 2	13.32 (0.23)	12.91 (0.42)	13.54 (0.43)	0.017 (0.002, 0.039)
*p*-value ^a^		N.S.	N.S.	

Model 1: Age and sex adjusted mean scores. Model 2: Age, sex, HUI-3, hearing device use period (years), and hearing loss severity (four-frequency average hearing loss). N.S. denotes a non-significant *p*-value, and ^a^ denotes significant *p*-values when compared to hearing aid users.

**Table 3:** Adjusted comparison of mean Attitudes towards Loss of Hearing Questionnaire (ALHQ) scores among hearing device users.

	Device User Groups			
Adjusted Mean Scores (Standard Error)	Hearing Aid	Cochlear Implant	Bimodal	Effect Size (Confidence Level)
**Total Score**				
Model 1	2.46 (0.02)	2.29 (0.03)	2.27 (0.03)	0.048 (0.021, 0.079)
*p*-value ^a^		<0.0001	<0.0001	
Model 2	2.43 (0.02)	2.34 (0.03)	2.30 (0.03)	0.044 (0.017, 0.077)
*p*-value ^a^		0.0801	0.0038	
**Denial Score**				
Model 1	2.08 (0.03)	2.08 (0.05)	1.79 (0.06)	0.031 (0.010, 0.057)
*p*-value ^a^		N.S.	<0.0001	
Model 2	2.06 (0.03)	2.12 (0.06)	1.95 (0.06)	0.037 (0.002, 0.039)
*p*-value ^a^		N.S.	N.S.	
**Negative Association Score**				
Model 1	2.24 (0.04)	1.74 (0.08)	1.84 (0.08)	0.032 (0.011, 0.059)
*p*-value ^a^		<0.0001	<0.0001	
Model 2	2.22 (0.05)	1.90 (0.09)	1.92 (0.09)	0.027 (0.007, 0.054)
*p*-value ^a^		0.0067	0.0145	
**Negative Coping Score**				
Model 1	2.94 (0.03)	2.85 (0.06)	3.01 (0.06)	0.005 (0.000, 0.018)
*p*-value ^a^		N.S.	N.S.	
Model 2	2.93 (0.04)	2.89 (0.06)	2.96 (0.07)	0.010 (0.000, 0.028)
*p*-value ^a^		N.S.	N.S.	
**Dexterity and Vision Score**				
Model 1	1.99 (0.04)	1.75 (0.08)	1.73 (0.08)	0.040 (0.016, 0.070)
*p*-value ^a^		0.0150	0.0158	
Model 2	1.94 (0.04)	1.95 (0.08)	1.81 (0.08)	0.047 (0.019, 0.080)
*p*-value ^a^		N.S.	N.S.	
**Esteem Score**				
Model 1	3.17 (0.05)	3.10 (0.09)	2.74 (0.09)	0.027 (0.007, 0.052)
*p*-value ^a^		N.S.	<0.0001	
Model 2	3.15 (0.05)	3.03 (0.10)	2.84 (0.10)	0.029 (0.008, 0.057)
*p*-value ^a^		N.S.	0.0199	

Model 1: Age and sex adjusted mean scores. Model 2: Age, sex, HUI-3, hearing device use period (years), and hearing loss severity (four-frequency average hearing loss). N.S. denotes a non-significant *p*-value, and ^a^ denotes significant *p*-values when compared to hearing aid users.

**Table 4:** Adjusted comparison of mean Speech, Spatial, and Qualities of Hearing Scale (SSQ) scores among hearing device users.

Device User Groups				
Adjusted Mean Scores (Standard Error)	Hearing Aid	Cochlear Implant	Bimodal	Effect Size (Confidence Level)
**Total Score**				
Model 1	4.66 (0.09)	4.37 (0.17)	3.87 (0.17)	0.026 (0.007, 0.051)
*p*-value ^a^		N.S.	<0.0001	
Model 2	4.58 (0.09)	4.49 (0.16)	4.22 (0.17)	0.033 (0.010, 0.063)
*p*-value ^a^		N.S.	N.S.	
**Speech Score**				
Model 1	3.78 (0.10)	3.86 (0.18)	3.36 (0.19)	0.007 (0.000, 0.021)
*p*-value ^a^		N.S.	0.1431	
Model 2	3.70 (0.10)	3.84 (0.18)	3.56 (0.19)	0.011 (0.000, 0.063)
*p*-value ^a^		N.S.	N.S.	
**Spatial Score**				
Model 1	5.21 (0.12)	4.16 (0.21)	3.79 (0.22)	0.068 (0.036, 0.103)
*p*-value ^a^		<0.0001	<0.0001	
Model 2	5.13 (0.12)	4.57 (0.22)	4.45 (0.23)	0.070 (0.035, 0.108)
*p*-value ^a^		0.0885	0.0254	
**Qualities Score**				
Model 1	5.35 (0.10)	5.16 (0.18)	4.55 (0.18)	0.023 (0.005, 0.047)
*p*-value ^a^		N.S.	0.0001	
Model 2	5.28 (0.10)	5.24 (0.18)	4.87 (0.18)	0.031 (0.009, 0.060)
*p*-value ^a^		N.S.	0.1410	

Model 1: Age and sex adjusted mean scores. Model 2: Age, sex, HUI-3, hearing device use period (years), and hearing loss severity (four-frequency average hearing loss). N.S. denotes a non-significant *p*-value, and ^a^ denotes significant *p*-values when compared to hearing aid users.

**Table 5:** Adjusted comparison of mean International Outcome Inventory for Hearing Devices (IOI-HD) scores among hearing device users.

	Device User Groups			
Adjusted Mean Scores (Standard Error)	Hearing Aid	Cochlear Implant	Bimodal	Effect Size (Confidence Level)
**Total Score**				
Model 1	27.18 (0.21)	29.92 (0.38)	29.28 (0.39)	0.071 (0.038, 0.107)
*p*-value ^a^		<0.0001	<0.0001	
Model 2	27.31 (0.22)	29.35 (0.40)	29.03 (0.41)	0.081 (0.044, 0.121)
*p*-value ^a^		<0.0001	0.0007	
**Factor 1**				
Model 1	16.16 (0.14)	18.65 (0.25)	18.32 (0.25)	0.136 (0.092, 0.180)
*p*-value ^a^		<0.0001	<0.0001	
Model 2	16.34 (0.15)	18.00 (0.27)	17.65 (0.27)	0.140 (0.093, 0.187)
*p*-value ^a^		<0.0001	<0.0001	
**Factor 2**				
Model 1	11.02 (0.12)	11.27 (0.21)	10.96 (0.22)	0.002 (0.000, 0.012)
*p*-value ^a^		N.S.	N.S.	
Model 2	10.97 (0.12)	11.34 (0.22)	11.37 (0.23)	0.005 (0.000, 0.019)
*p*-value ^a^		N.S.	N.S.	

Model 1: Age and sex adjusted mean scores. Model 2: Age, sex, HUI-3, hearing device use period (years), and hearing loss severity (four-frequency average hearing loss). N.S. denotes a non-significant *p*-value, and ^a^ denotes significant *p*-values when compared to hearing aid users.

## Data Availability

The data that support the findings of this study are available from the corresponding author upon reasonable request. The data underlying the results in the study are considered highly sensitive as they involve the health status of adults with hearing loss. Even when de- identified, there is potential for harm, and this will be considered when assessing requests for data sharing.

## References

[1] Wang J., Puel J.L. (2020). Presbycusis: An Update on Cochlear Mechanisms and Therapies. J. Clin. Med..

[2] Mick P., Parfyonov M., Wittich W., Phillips N., Pichora-Fuller M.K. (2018). Associations between sensory loss and social networks, participation, support, and loneliness: Analysis of the Canadian Longitudinal Study on Aging. Can. Fam. Physician.

[3] Jayakody D.M.P., Wishart J., Stegeman I., Eikelboom R., Moyle T.C., Yiannos J.M., Goodman-Simpson J.J., Almeida O.P. (2022). Is There an Association Between Untreated Hearing Loss and Psychosocial Outcomes?. Front. Aging Neurosci..

[4] Tseng Y.-C., Gau B.-S., Liu T.-C., Hsieh Y.-S., Huang G.-S., Lou M.-F. (2022). Association between sensory impairments and restricted social participation in older adults: A cross-sectional study. Collegian.

[5] Livingston G., Huntley J., Liu K.Y., Costafreda S.G., Selbæk G., Alladi S., Ames D., Banerjee S., Burns A., Brayne C. (2024). Dementia prevention, intervention, and care: 2024 report of the Lancet standing Commission. Lancet.

[6] Choi J.S., Adams M.E., Crimmins E.M., Lin F.R., Ailshire J.A. (2024). Association between hearing aid use and mortality in adults with hearing loss in the USA: A mortality follow-up study of a cross-sectional cohort. Lancet Healthy Longev..

[7] Zeitler D.M., Prentiss S.M., Sydlowski S.A., Dunn C.C. (2024). American Cochlear Implant Alliance Task Force: Recommendations for Determining Cochlear Implant Candidacy in Adults. Laryngoscope.

[8] Tang D., Tran Y., Lo C., Lee J.N., Turner J., McAlpine D., McMahon C., Gopinath B. (2024). The Benefits of Cochlear Implantation for Adults: A Systematic Umbrella Review. Ear Hear..

[9] Tang D., Tran Y., Bennett R.J., Lo C., Lee J.N., Turner J., Gopinath B. (2025). The Benefits of Hearing Aids for Adults: A Systematic Umbrella Review. Ear Hear..

[10] Ching T.Y., Leigh G., Dillon H. (2013). Introduction to the longitudinal outcomes of children with hearing impairment (LOCHI) study: Background, design, sample characteristics. Int. J. Audiol..

[11] Bisgaard N., Zimmer S., Laureyns M., Groth J. (2022). A model for estimating hearing aid coverage world-wide using historical data on hearing aid sales. Int. J. Audiol..

[12] Sorkin D.L., Buchman C.A. (2016). Cochlear Implant Access in Six Developed Countries. Otol. Neurotol..

[13] Tang D., Tran Y., McMahon C., Turner J., Amin J., Sinha K., Alam M.N., Wuthrich V., Sherman K.A., Garcia P. (2023). A protocol for the Hearing impairment in Adults: A Longitudinal Outcomes Study (HALOS). PLoS ONE.

[14] Horsman J., Furlong W., Feeny D., Torrance G. (2003). The Health Utilities Index (HUI): Concepts, measurement properties and applications. Health Qual. Life Outcomes.

[15] Tomioka K., Ikeda H., Hanaie K., Morikawa M., Iwamoto J., Okamoto N., Saeki K., Kurumatani N. (2013). The Hearing Handicap Inventory for Elderly-Screening (HHIE-S) versus a single question: Reliability, validity, and relations with quality of life measures in the elderly community, Japan. Qual. Life Res..

[16] Faraji-Khiavi F., Bayat A., Dashti R., Dindamal B., Ghorbani Kalkhajeh S. (2023). Consistency of two versions of hearing handicap inventory for elderly (HHIE and HHIE-S) with degree of hearing loss (HL). Hear. Balance Commun..

[17] Noble W., Jensen N.S., Naylor G., Bhullar N., Akeroyd M.A. (2013). A short form of the Speech, Spatial and Qualities of Hearing scale suitable for clinical use: The SSQ12. Int. J. Audiol..

[18] Cox R.M., Alexander G.C. (2002). The International Outcome Inventory for Hearing Aids (IOI-HA): Psychometric properties of the English version. Int. J. Audiol..

[19] Saunders G.H., Jutai J.W. (2004). Hearing specific and generic measures of the psychosocial impact of hearing aids. J. Am. Acad. Audiol..

[20] Kim A.S., Betz J.F., Nieman C.L., Hoyer M.R., Applebaum J., Lin F.R., Goman A.M. (2021). Long-Term Impact of Hearing Aid Provision or Cochlear Implantation on Hearing Handicap. Laryngoscope.

[21] Lim S., Turner J., Tang D., Sherman K., Sinha K., Chawla S., Carney S., Shekhawat G.S., Gopinath B. (2025). Understanding hearing health-care access in Australia: Users’ perspectives. Australas. J. Ageing.

[22] Ruusuvuori J.E., Aaltonen T., Koskela I., Ranta J., Lonka E., Salmenlinna I., Laakso M. (2021). Studies on stigma regarding hearing impairment and hearing aid use among adults of working age: A scoping review. Disabil. Rehabil..

[23] Howard J.S., Mattacola C.G., Howell D.M., Lattermann C. (2011). Response shift theory: An application for health-related quality of life in rehabilitation research and practice. J. Allied Health.

[24] Oosthuizen I., Manchaiah V., Launer S., Swanepoel W. (2022). Hearing aid Experiences of Adult Hearing aid Owners During and After Fitting: A Systematic Review of Qualitative Studies. Trends Hear..

[25] Gatehouse S., Noble W. (2004). The Speech, Spatial and Qualities of Hearing Scale (SSQ). Int. J. Audiol..

[26] Shehorn J., Marrone N., Muller T. (2018). Speech Perception in Noise and Listening Effort of Older Adults with Nonlinear Frequency Compression Hearing Aids. Ear Hear..

[27] van Wieringen A., Magits S., Francart T., Wouters J. (2021). Home-Based Speech Perception Monitoring for Clinical Use with Cochlear Implant Users. Front. Neurosci..

[28] Wu X., Ren Y., Wang Q., Li B., Wu H., Huang Z., Wang X. (2019). Factors associated with the efficiency of hearing aids for patients with age-related hearing loss. Clin. Interv. Aging.

[29] Gopinath B., Shineh G., Tang D. (2026). From Hearing Aid Uptake to Hearing Aid Effectiveness: Rethinking Dementia Risk Reduction in Adults With Hearing Loss. Sens. Neurosci..

[30] Jiang F., Dong Q., Jayakody D.M., Chen X., Mueller C., Wu B., Underwood B.R., Yan L.L., Li X., Bruck C. (2026). Hearing aid effectiveness and probable dementia risk across 33 countries: A pooled analysis of seven cohorts. Cell Rep. Med..

[31] Han J.S., Lim J.H., Kim Y., Aliyeva A., Seo J.H., Lee J., Park S.N. (2024). Hearing Rehabilitation With a Chat-Based Mobile Auditory Training Program in Experienced Hearing Aid Users: Prospective Randomized Controlled Study. JMIR mHealth uHealth.

[32] Graham M.A., Nicholson N., Glade R., Delport S., Mahomed-Asmail F. (2025). Person-centered care in adult auditory rehabilitation: A scoping review. Int. J. Audiol..

